# Should I Stay or Should I Go? The Impact of Social Networks on the Choice to Play for a National Team in Football

**DOI:** 10.3390/ijerph18157719

**Published:** 2021-07-21

**Authors:** Klaus Seiberth, Ansgar Thiel

**Affiliations:** 1 Faculty of Management, Economics and Social Sciences, Institute of Sports Science and Kinesiology, University of Stuttgart, 70569 Stuttgart, Germany; 2 Faculty of Economics and Social Sciences, Institute of Sports Science, University of Tübingen, 72074 Tübingen, Germany; a.thiel@uni-tuebingen.de

**Keywords:** elite sport, youth elite football, migrant background, national team, network theory, social network analysis

## Abstract

In the course of their careers, elite athletes are faced with crucial decisions. This applies particularly to adolescent athletes who additionally have to cope with a variety of age-related developmental tasks. For young top football players with a migrant background, this can be even more challenging as they often attract the interest of national associations. From a network-theoretical perspective, it can be considered likely that the decision to join a top national association is not taken independently of the players’ networks. This article addresses the role of network actors within the players’ decision-making process. Our analysis is guided by constructivist network theory and based on a qualitative research approach that used guided expert interviews as its core research tool. Ten interviews with German-born youth internationals with a migrant background were conducted. The present analysis reveals several network actors such as family, coaches and players’ agents involved in the ‘national team question’. Evidently, most relevant networks of players with a migrant background are sports-related. These networks turned out to be highly functionalized and leave only limited room for manoeuvring. At the same time, the interviews reveal ‘structural holes’ within the players’ networks and indicate a considerable need for the optimization of talent counselling.

## 1. Introduction

According to the German Federal Statistical Office more than 25% of the German population currently has a migrant background. Having a migrant background means that a person “or at least one parent did not acquire German citizenship by birth” [1]. In German professional junior football players with a migrant background are highly represented. Most of these players are born in Germany. They join the talent development programme of the German Football Association (DFB), play in the junior teams of professional German football clubs and train at their Youth Performance Centres (YPC). The top talents among these adolescent athletes are confronted with a special situation: in contrast to their teammates without a migrant background, they can attract the interest of two different top national associations. The example of German national players with a Turkish background—such as Mesut Özil, Ilkay Gündogan or Emre Can—illustrates that both the German Football Association (DFB) and the Turkish Football Federation (TFF) usually put feelers out for the athletes at a fairly early stage of their junior careers. On top of the high demands of junior elite football and school, and in addition to a number of age-related developmental tasks, these players have to deal with the question of which national team they want to play for. This can be challenging for the adolescent players, as the decision of signing up (which is still reversible at the junior level) not only concerns questions of ethnic or national affiliation but can also have a considerable impact on their sporting careers [2]. 

In the case of top adolescent athletes, one can assume that the decision to join a top national association is not made completely autonomously. These athletes are surrounded by a network of different actors who provide support in the pursuit of sporting success [3]. Accordingly, it can be considered likely that the players address the ‘national team question’ within their networks. It stands to reason that such networks also consist of players from the top sporting environment, as the players are included in federal talent promotion structures such as Youth Performance Centres (YPC) (In German: Nachwuchsleistungszentrum) and are, therefore, in close contact with fellow players, coaches, youth coordinators and pedagogical staff. Recruitment for the regional football associations’ state teams and the DFB’s national youth teams means that further officials, coaches and players enter the stage who can influence the players and their decision-making process with regard to the national team question. In addition, many of these highly talented junior players are already counselled by players’ agents and agencies. Next to this top sport-related environment, the junior athletes have relationships with their families, teachers as well as peers outside of top sports (friends, classmates), which indicates how many environments and network actors can be involved in career-relevant decisions. In view of the complexity of network relations, the question of the role the various network actors play in the decision-making process surrounding the national team question is all the more interesting. Players are not just athletes being forced to take career-relevant decisions. They are also adolescents with a migrant background who must cope with a number of age-related developmental tasks. During this crucial stage of personal development and elite sports careers, these players increasingly depend on the professional and emotional support of network actors.

A review of the state of research shows that there are various current studies that focus on decision-making processes by using complex network analytical models. Most of these works are based on a quantitative paradigm by analysing the impact of networks and relationships on decision-making [4,5,6]. Within the last few years, sports research in general, and elite sport research in particular, have produced several works based on network approaches that analyse specific network constellations in (elite) sport [7,8,9]. These works have significantly contributed to establishing a sport-related social network research base. Among these studies, however, there are only a few that refer to decision-making processes or address the role of network actors in the field of (elite) sports [10,11,12]. It is evident that these works do not refer to elite youth sports and their specific context constellations nor to athletes with a migrant background as a specific population group. In fact, the impact of actors from the environment on career-relevant decisions in youth football has barely been addressed in national and international research to date, despite a series of interesting studies on the connection of migrant backgrounds, social identities and national team careers from the players’ perspective [2,13,14,15]. Accordingly, little is known about the environment and network actors involved in career-relevant decision-making processes in top junior sports, the role they play in this process and the way junior athletes process the various actors’ input. This research gap is very surprising in view of the high number of young talented athletes with a (Turkish) migrant background in German youth football (as illustrated by the YPC’s ‘squad lists’ and the German national youth teams). 

Against this backdrop, we address the significance of surrounding actors in the process of deciding on a top national association, particularly focusing on the network relationships relevant for this process, thus adopting a perspective that has been largely neglected in (German) sports research so far [7]. 

The study intends to “further the agenda of social network analysis” [16] (p. 25) in the field of (youth) elite sport and migration research. In addition, the study aims to contribute to talent promotion by identifying potentials for improvement in elite youth football. This article is based on a comparative qualitative study consisting of ten guideline-based expert interviews with German junior national players with a Turkish background.

## 2. Theoretical Background

In order to render network relationships between players and relevant actors in the environment and be able to analytically consider the subjective processing of network relationships’ and environmental influences, we base our analysis on a network-theoretical perspective.

### 2.1. Network Theory

Our analysis is guided primarily by constructivist network theory, which focuses on the structure and quality of relationships between different actors [17,18,19]. Network theories assume that individuals “cannot be considered in isolation from their networks” [20] (p. 50). The perspective of network research, as Harrison White puts it, leads away “from individuals or elements as the unit of analysis and (puts) relations (social relationships) in their place” [21] (p. 387). In this sense, networks are understood as a “web of social relations in which individuals, collectives or corporate actors are embedded” [22] (p. 188). In contrast to classical social-structure analysis, which focuses primarily on individual characteristics (such as gender, age, ethnic origin, etc.) [20], network analyses thus examine aspects of relationality or, more precisely, the “interrelational linking of relationships and social contexts with each other” [23] (p. 245). Accordingly, focusing on social networks implies a “fundamental shift from traditional approaches in social sciences focusing on attributive categories of individuals to relational processes or transactions between individuals” [7] (p. 139). From the perspective of constructivist network analyses, actors process-relational influences subjectively, i.e., they are not passive transformers of environmental and network influences, but rather co-design their networks and autonomously give meaning to the impulses passed on via network relationships [24,25].

In the present study, we assume that the decision to join a national team, like social action in general, is considered “highly dependent on the relational structure (assumed to be relatively stable) between actors” [26] (p. 117). So-called “sociograms” can be used to examine the relationship structure between the relevant actors [18,27]. Networks are represented as social structures here, consisting of actors (“nodes”) and their relationships to each other (“ties”). Through these “ties”, people or groups enter into exchanges with each other [7,25]. Networks are conceptualised as “exchange systems” oriented towards the “circulation of resources and information” [26] (p. 119).

Sociometry visualises network structures with the help of tools, e.g., “graphs” and “nodes”. Contact intensity and the strength of the connection [17,19,28] are of great importance. In network research, a distinction is made between “strong ties” and “weak ties” [17]. Weak ties are characterised by low contact frequency and low intimacy. Strong ties, on the other hand, exist between people who have a high frequency of contact and maintain an intense emotional relationship. Such close ties often exist with family members. Especially in adolescence, relationships with peers are intensified and function as strong, emotionally supportive ties [17,25].

### 2.2. Youth Elite Sport as a Network

For (junior) elite sport research, network analyses offer the advantage of revealing the “interrelational linking of relationships and social contexts” [23] (p. 245) and thus rendering the special features of relationship constellations in the setting of (junior) elite sport visible. One special feature of network structures in top youth sport, for example, is that many highly talented youth athletes in football move to YPCs at an early stage in their biographies and have to leave their familiar (family) environment in the process. Such a change of social environment usually starts a process of “hyper-inclusion” [29] (p. 111), with the athletes increasingly entering an “identity tunnel” [30] (p. 280). In the research literature, this process is also described as the “biographical trap” of elite sports [3] (p. 39), since the increasing commitment to the elite sport is accompanied by decreasing contact frequency and intensity with regard to family and friends, while at the same time increasing the quantity and quality of social contacts with elite sports actors such as coaches [31]. It is precisely in this phase of increasing biographical fixation on an elite sport that the national team question often arises for the first time.

From a sociological perspective, the fact that sport-related networks have “structural holes” [32,33] resulting from incomplete relationship constellations or missing relationships bears relevance [20] (p. 55). While the concept of weak and strong ties looks at the intensity of relations, the concept of structural holes focuses on the structure and the position of actors within a network. Structural holes are significant in that they can prevent the exchange of relevant information. Important options for action and decision-making are thus not available to the decision-maker. In order to plug structural holes in networks, actors are needed that have specific decision-relevant knowledge and are able to share this with the decision-maker [32,34]. Such actors are referred to as “brokers” in network theory. The specific position of these brokers is based on an “information arbitrage” [32] (p. 354) and allows them to “bridge the structural holes between groups [and] have an advantage in detecting and developing rewarding opportunities” [32] (p. 354). Coaches or players’ agents may take broker positions in the decision-making process of junior players when they provide players with specific information about options regarding the national teams’ issue or specify career prospects associated with the decision. Broker functions can also be taken on by emotionally close people. With regard to the social networks of people and athletes who have a Turkish background, the universalist idea that relationships with the family of origin, in particular, are shaped in a special way by “culturally” conditioned expectations and a strong ethnic and national identity [35,36], is supposed to be the main factor for the team decision of players with Turkish background [37]. Universalistic approaches assume that in the environments of young German-born athletes with a Turkish background, there are actors who instrumentalise the decision, e.g., by making the national team question a decision of “honour”, ethnicity or national attachment to the parents’ or grandparents’ country of origin. 

In principle, attribution to a social network is always linked to network-related “stories”, whose central themes represent collective experiences the actors share with each other and through which they relate to each other [20] (p. 52). Such network-related stories can also be found in connection with the decision-making process regarding the country or association a young player with a migrant background will play for. In these stories, sports associations play a central role as network actors by extending invitations to players to participate in training courses or international matches and informing the clubs or youth centres about such invitations. At the same time, however, these stories (in addition to the distinction of “invitation”) also contain stereotypical, culturalising representations of cultural affiliation or family and national expectations [2]. 

In conclusion: Network analyses open up a highly interesting perspective with regard to the national team issue among young players with a migrant background. This also applies when considering that network-theoretical analyses of career-relevant athlete decisions are not yet available. With the choice of this network-theoretical perspective, the player, in their relationships to the relevant environment or network actors, moves to the centre of attention. The focus, on the one hand, lies on the identification of relevant actors who are communicatively involved in the decision-making process, and on the other hand, on the quality of the (exchange) relationships with the various actors. The main question here is which actors are involved in the decision-making process and how they influence the player. Looking at these network structures also enables the identification of structural holes and/or so-called “brokers”.

In the relational perspective of network research, the focus is on the young athlete in his exchange relationships with his environment. In this context, he is both an indispensable “agent of top-level sport” [3] (p. 143) and a central decision-maker in the national team issue. In this sense, the following analysis looks at the player from an “ego-centred” perspective, the network’s core individual as it were [25,38].

## 3. Materials and Methods

The study is based on a qualitative research approach that aimed to achieve three objectives. The first objective was to reconstruct the decision-making process in choosing to join a national team and to identify relevant reasons that determine the decision. Based on the media and public discourse that assumes ethnic identification as the key factor within the national team question, the study secondly intended to reflect the role of ethnic identity for the choice from a players’ perspective. Thirdly, the study aimed to investigate the role of relevant network actors in this decision-making process. The present article focuses on the third objective and presents data about this topic.

Based on our theoretical network perspective, we assumed that individuals (at least partially) shape their networks themselves and constructively deal with the challenges and expectations relevant network actors explicitly or implicitly place on them in the course. For this reason, we used a method for collecting data that, on the one hand, makes it possible to identify relevant network actors with regard to the national team issue from the player’s point of view and to document the change in the networks, and, on the other hand, to make the subjective processing of these influences the object of study. 

Although field access to top-level football is very difficult, we succeeded in conducting ten case analyses with young athletes with a Turkish background from top-level German football. There were two reasons why we exclusively concentrated on this specific ethnic group. The first reason was that the ‘German-Turkish’ community is the largest immigrant population in Germany. The second reason was that the public and media debate on the national team question in Germany almost exclusively refers to players with Turkish backgrounds—such as Mesut Özil or Ilkay Gündogan [2].

In order to find potential interviewees, we started a web search using the online database www.transfermarkt.de (accessed on 24 May 2021). This website provides a wide range of relevant data on elite (junior) football players in German (junior) teams. By focusing on the feature ‘nationality’, we identified numerous top-level players who were characterised as being both ‘German’ and ‘Turkish’. The case selection was based on the following criteria: The players had to have a Turkish background, be between 15 and 21 years old, have been born in Germany and be trained at a German YPC. A further essential requirement was that the players had played at least one international match for the Turkish Football Federation or the German Football Association in the course of their career. After we identified 31 players who matched the selection criteria, we officially contacted the YPCs in their function as gatekeepers. We informed them about the aim of the study, the specific target group and the players we aimed to recruit. The YPCs examined our request and autonomously decided whether to inform the respective players and their parents of our request. In case of a positive assessment, we received either date proposals for the interviews or the contact details of the players in order to arrange an interview on-site. This way, we respected the responsibilities of the YPCs and avoided ethical concerns by directly influencing the players in their decision to participate in the study. During this recruitment and termination process, we were confronted with typical problems researchers face when trying to recruit (youth) elite athletes for research purposes.

Nevertheless, several YPCs, and ten players, responded positively to our request. The interviews with these ten players were conducted between 2013 and 2015. In order to keep players’ time expenditure to a minimum and to use familiar surroundings, the interviews were conducted at the players’ club facilities. Since the players were born and socialised in Germany, the interviews were carried out in German. All interviewees spoke German fluently. In order to avoid a systematic distortion of results due to sampling bias, we selected players from different groups. The sample consisted of youth players who had either played for the German Football Association (DFB) or the Turkish Football Federation (TFF) only. Moreover, we recruited players who had played for both federal football associations (DFB and TFF) during their youth career—which was in accordance with FIFA’s eligibility regulations [39]. The interview duration was between 45 and 90 min. Transcribed verbatim in German, key statements were translated into English by the authors and checked by a professional translator.

Interviewing young people requires specific reflections on ethical issues. We, therefore, made transparency, confidentiality and balanced power relations our highest priorities. According to the ethical standards of qualitative research, our study was based on the concept of informed consent. Participation in the study was thus entirely voluntary. Confidentiality and anonymity were guaranteed to interviewees and their clubs. In order to encourage the players to express their views freely and avoid socially desirable answers, we accentuated the fact that we were not interested in any kind of moral assessments regarding the players’ choice.

Guided expert interviews [40] represented the core of the research instrument. The interviews were first recorded and transcribed before we conducted a content analysis and a comparative analysis. The process of deciding to join a national team, the role of the surrounding actors involved and the processing of network influences by the young athletes with a migrant background were to be reconstructed with the help of the interviews. In an initial step, we created case profiles to summarise relevant contextual information. In the next step, we coded the text thematically in order to identify ‘content analytical units’ [40]. During this process of categorizing and sorting data, we identified main categories, statements, and themes using MAXQDA software. In a final step, we prepared case studies in order to systematically compare the cases by identifying patterns of similarity and difference.

The case studies were less concerned with mapping networks in their entire complexity in the sense of sociometry. Rather, the focus was primarily on analysing the “story” of the process of deciding in favour of a national team, secondly on identifying the surrounding actors with whom this story was shared and thirdly on investigating the ties to these actors and the relevance of these connections with regard to the decision. Particular attention was paid to the identification of structural holes or broker positions in the networks. The biographical perspective allowed us to trace the change of networks regarding the national team question from the players’ points of view.

## 4. Results and Discussion

Only a small number of actors are mentioned as relevant for the decision-making process in the players’ accounts. Strikingly, similar network constellations are found in most of the case studies. Three network actors, in particular, appear prominently: parents and family members, coaches, and players’ agents. Considering the increasing “biographical fixation” [3,31] on (youth) elite sport, it is not surprising that various elite sports actors are mentioned within the players’ reconstructions. Across the different interviews, it became evident that the actors consider the youth athlete the decision-maker. Although the interviews show that the environmental actors undoubtedly exert influence on the youth athletes as well as the decision-making process, the players point out that the actors “leave” the decision to them as elite athletes:

“Yes, and then they just left the decision to me and said: You are the player, it’s your decision, and then I just decided to play for the Turks.”

(Interviewee 2, Youth International TFF)

### 4.1. Parents, Family and Peers

On the part of the non-athletic environment, it is especially parents and family members who are actively involved by the players in the career-relevant decision-making process and who appear as central contact persons. Fathers often play a central role. However, the fact that family or family members comment on the national team issue neither means that they are assigned decisive influence on the content of the decision nor that they represent unambiguous or similar positions. Parents and other family members (e.g., grandparents) appear in the interviews primarily as emotional supporters. The players emphasise that their parents do not want to push their sons to make a certain decision.

“Generally, I discuss everything with my family. But basically, in the end, they leave the decision up to me.” (Interviewee 3, Youth International TFF)

Instead, the players regard themselves as independent and competent decision-makers in their relationships with their parents.

“My father supports me in every way. He told me to make up my mind. And he asks, he tells me what’s coming and then he tells me what he thinks, but in the end I decide. So, of course my family tells me what they advise. But in the end I have to decide what’s best for me.”

(Interviewee 7, Youth International TFF)

“So they also left the decision up to me. They said: ‘You can decide for yourself because in the end it’s you who’s playing.’ And yes, that was good, so I wouldn’t let others tell me where to play, either. That’s important for me. And in the end, as my parents have already said, I am the one who is playing and so I also get to decide.”

(Interviewee 8, Youth International DFB)

A characteristic of this exchange relationship is that the young athletes experience a high degree of appreciation and recognition from the family and especially from their parents for what they have achieved athletically—regardless of whether the invitation is extended by the German, the Turkish or both associations.

“They also asked: Did you get an invitation? [...] They were happy that I got an invitation. [...] What else is there to discuss then.”

(Interviewee 1, Youth International DFB & TFF)

“The [family and relatives] were just as proud when I then played in the German national team. That was not the question at all. They’re happy about everything I’ve achieved, they’re really happy with me.”

(Interviewee 4, Youth International DFB & TFF)

Despite the primary appreciation of the athletic aspects, the national team issue seems to be thoroughly emotional in some families.

“My father and mother wanted me to play for Turkey because there is a bit of pride and honour somehow and this is very important for the Turks.”

(Interviewee 2, Youth International TFF)

However, even in these cases, emotional considerations appear mixed with more practical athletic considerations regarding the players’ career prospects.

“My uncle thought differently to them, he thought more about my future. And he said: ‘You’d better play for the Germans.’”.

(Interviewee 2, Youth International TFF)

Thus, the national team question is discussed controversially within the family in that, on the one hand, it is symbolically charged as a decision between the parents’/grandparents’ country of origin and the young athlete’s country of birth. On the other hand, however, it is identified as a purposeful and athletic decision based on existing career options as well as prospects of success and future prospects. This is in accordance with various findings in elite sport research. Being socialised and hyper-included into elite youth football means that athletes internalise the dominant logic of elite sport and integrate this logic into their own decision-making [31]. The interviews show that this also applies to the national team question [2].

It should be noted, however, that the national team question is only emotionalised in connection with the parents’ or grandparents’ country of origin (Turkey), while the potential choice for the young athletes’ country of birth is not emotionally symbolically framed but attributed in terms of athletic performance.

“So my father always said: ‘If you want to be successful, play for the German [national team] and if you want to play for your fatherland, play for the Turkish [national team].’”

(Interviewee 6, Youth International TFF)

However, this in no way means that a career in the German national team, and thus, the youth athletes’ country of birth would be devalued by the parents or family.

As a rule, the junior athletes perceive influences from the family environment neither as problematic nor as irritating with regard to their own considerations. Accordingly, family support has no ultimate relevance beyond emotional backing with regard to the decision itself.

“Actually–to be honest—my [parents] wanted me to play for Germany, but if I don’t play there, why should I not play for Turkey instead?”

(Interviewee 1, Youth International DFB & TFF)

These results are particularly interesting against the background of Granovetter’s concept of strong and weak ties [17]. Given the parents’ merely secondary relevance in the actual decision-making process regarding the national team question, they represent only weak ties in the players’ social network from an athletic point of view, although they can certainly be considered strong ties from an emotional perspective. Even if young athletes identify parents and family as central emotional reference persons, it should be borne in mind that the majority moved to YPC boarding schools at a very early stage, which also reduced the frequency of contact with their families. Accordingly, in the everyday lives of these top adolescent athletes, there are contact-intensive relationships mostly with players from the top sports (club) environment (above all with club coaches or other YPC staff). The determination of the quality of relationships between parents and children in elite youth sport is, therefore, less clear-cut than assumed in network theories. Most likely, the relationship with family members tends to develop into weak ties in the process of hyper-inclusion in elite sport, since the elite sports career and the coupling of school and elite sport tie up substantial time resources, leaving little room for cultivating other relationships. 

Apart from family members, hardly any other actors from the non-athletic environment appear in the players’ network descriptions. The absence of friends is surprising since friends are described in socialisation theory as actors with whom adolescents usually have close relationships (strong ties), not least with regard to coping with the developmental task of detachment from the parental home [41]. 

When friends are referred to in the interviews, it is usually in the context of friends benefitting from the image transfer of a call-up to the national team themselves.

“They supported me every time, regardless of whether it was with Turkey [or Germany]—I took pictures, sent them pictures, and of course they thought it was really cool.”

(Interviewee 4, Youth International DFB & TFF)

In contrast to parents, however, friends are not marked as relevant network actors in an emotional sense. One player reported that he did not even tell his friends from his former place of residence about the Turkish federation’s invitation to avoid the news spreading in his hometown.

“I didn’t talk about it with my mates because I didn’t want it to get around in [place name], for example, around town, which isn’t that big, and then it would get around everywhere.”

(Interviewee 2, Youth International TFF)

These findings need to be discussed against the background of elite sport socialisation [3,30,31]. Accordingly, the marginal role of peers in the players’ social networks can be explained with regard to the competitive sports socialisation of young athletes. For highly talented young football players (irrespective of possible migrant backgrounds), detachment from the parental home is virtually inevitable, as they have to leave their primary environment during adolescence and move to boarding schools at YPCs. In this sense, young athletes do not need additional socialisation agents to support them in this youth-specific developmental task. Furthermore, they are in a competitive relationship with other peers, such as teammates. On the one hand, they are in daily contact with their teammates, spend a lot of time and cooperate with them. On the other hand, teammates are also rivals, who compete for the same scarce resources such as playing time, squad places or regular spots. Building relationships of trust like the ones players may have cultivated with friends before their competitive sports careers is likely difficult under these conditions.

### 4.2. Coaches and Sport Associations

In the world of young athletes—which is dominated by elite sport—coaches have a considerable influence on the career and performance development of young athletes as athletic decision-makers. As a result of the high training volumes, the relationship between young athletes and club coaches is characterised by a high frequency of contact. Strong ties undoubtedly exist between young athletes and club coaches, accordingly. However, the relationship is asymmetrical in that the coach holds a central position of power with regard to the distribution of central resources (such as regular places, playing time, substitutions, etc.).

The club coaches are informed about invitations to their players through the formal communication channels between the association and the club or the YPC [42], and they usually know in advance about upcoming invitations to training courses, international matches or tournaments [2]. In the majority of cases, however, it is the players themselves who talk to the club coaches as soon as they receive an invitation to a training and recruitment course organised by a national football association. 

For some players, talking with the club coach about the invitation to a national team is very important in the run-up to the decision. While some club coaches initially express appreciation, assure support and encourage the players to continue and accept the invitation from the association, others take a clear position and argue primarily as representatives of the club/YPC and as representatives of the DFB’s talent development programme. With regard to the merits of being considered for a national team, it is evident that invitations and appointments by the Turkish Federation are valued less highly by some coaches than invitations from the German Football Association. In some cases, club coaches even tried to influence the decision by advising against the TFF and pointing out potential negative consequences (e.g., loss of regular place), which accepting the invitation from the Turkish Federation could entail for the players.

“Well, the first thing I did was go directly to my [club] coach. So I just asked: ‘Well, coach, I got an invitation. What‘s the deal? Should I go? What should I do?’ And at that time my coach said: ‘Well, you don’t even need to bother going. There’s no point in your case.’ So he only said negative things. I shouldn’t bother trying. (...) And I just said: ‘Well, why shouldn’t I go? If I have the chance, I want to take it.’ And he just said: ‘Well, the players I had before also went.’ The Turkish players he had as a coach. ‘And they didn’t make it.’ And then I said to him: ‘Yes, okay, but if they don’t make it, that doesn’t mean that I won’t make it either.’ And then he said: ‘Yes, it’s your decision. If you come back, you might not play or this and that.’ So they did threaten me with things like that. And then I just said: ‘No, I’ll go. I’ll give it a shot. And if I make it, then I make it. And if I don’t, then I’ll have just gained the experience of what it’s like there.’”

(Interviewee 5, Youth International TFF)

The players take note of these assessments by their club coaches but do not necessarily adopt them. Even though club coaches seem to be important contact persons, they do not have a determining role. Instead, the players process these influences productively by considering their advice from their own perspectives as players, which includes reflecting on the available options (e.g., the interest of one association or both) and anticipating individual deployment and career opportunities with the respective association. This finding is consistent with a constructivist network perspective assuming that actors process-relational influences subjectively and give meaning to these relational impulses [24,25].

When comparing the relevance of club coaches to that of national youth team coaches regarding the decision to play for a certain association, the national coaches are considered more influential, although the club coaches are closer to the players’ everyday lives:

“The thing that was very decisive for me was the coach of the German [U-]national team. (...). I had a conversation with the German national coach, a very long conversation actually, and he actually made it clear to me that I should not look at my career, but rather at what my heart says. And because I had been on both training courses, the decision was made relatively quickly.”

(Interviewee 3, Youth International TFF)

“He [the DFB’s national U team coach] definitely said that there are a lot of players who take advantage of this opportunity and first go back and forth and play a few games with Germany and then play a few games with Turkey. (.) He advised me not to do that because it would make a bad impression later on (-). He said that I should only listen to my heart and my feelings, and that’s how I decided in the end.”

(Interviewee 3, Youth International TFF)

Another player referred to the special relationship with the DFB’s national youth team coach at the time, which seems to have played a central role in the decision to switch from the TFF to the DFB. The national team coach appears in the players’ account as a “relationship promoter” [43] who is a representative of the DFB but also emotionally close to the players:

“And then [name of the DFB national youth team coach] called me in especially for the selection course because I had somehow said something to his secretary because I was also with Turkey and that it’s better here [at the DFB]. And then he said: ‘But one thing has to be clear, if I get you now, you’ll stay with me’, and so on. So that was a cool conversation he had with me. But, so then it was clear to me for the first time that I would be playing for the Germans. [Name of the coach] is a very good coach and also outstanding as a person, and the way he talked to me, it was already clear to me that I would be playing for him.”

(Interviewee 4, Youth International DFB & TFF)

Some players reported that coaches for the Turkish national U-team tried to influence the players during training courses or U international matches by threatening that accepting an invitation from the DFB would mean they would no longer be considered by the TFF. Whether these were personal strategies on the coaches’ parts or a TFF strategy cannot be reliably determined on the basis of the material.

“Last year, after the European Championship qualifiers [TFF], we were at the airport and our Turkish national team coaches, I don’t know how, had heard that two or three players had been invited [by the DFB]. So, the coach said, but I also thought he was right: ‘Whoever goes there won’t be invited to the Turkish national team because you all think we’re only second choice and we don’t need players like that.’ [...] I thought he was right. Then, I think two players left anyway. They weren’t invited back for a while, but they were invited back the other day.”

(Interviewee 1, Youth International DFB & TFF)

Considering the fact the national football associations’ development measures, training courses and youth international matches do not take place often, and the contact between players and national team coaches is rather sporadic or temporary [2,42], the connection between players and national coaches can be defined as weak ties. The fact that the association coaches’ influence is nevertheless marked “decisive” for the decision-making process can be explained by the fact that national team coaches are central decision-makers at the national team career level. Since they have power and decision-making authority, it is functional in terms of top-level sports logic to seek dialogue with them and to reflect on their assessment. Hence, the quality of the relationship between coach and player does not necessarily mirror its relevance with regard to career-related decisions. 

In the reconstructions of the decision-making process, corporate actors such as the YPC, the club or national football associations were considered functional network actors of a top-level sporting environment. Particularly YPCs were mentioned with regard to career-related decision-making processes by young elite players. YPCs have the power to intervene when, for example, the participation in training courses, international matches or tournaments is blocked by the school for reasons such as tests or exams, which happens every now and then, despite agreements between schools and the association.

“I was often not sent to the invitations sometimes. So I wasn’t allowed to go sometimes. Because they said: ‘Yes, okay, you have (-) if you left now, then if you came, you wouldn’t play. And (.) there were just a lot of problems with me because I’m with the Turkish national team.”

(Interviewee 5, Youth International TFF)

“At school, it was just that I (-) had an extremely large number of international matches for a while. And the school didn’t want to let me go, which I understand. But I was at a cooperation school, which [club name] is associated with, and they often said: ‘Well, it doesn’t make sense for you to go. Because of course you have a lot of times of absence. And sometimes they caused problems. But otherwise not.”

(Interviewee 5, Youth International TFF)

Here, the YPC emerges as a network actor that can use its power to influence the decision-making process. In this context, it should be noted that in the few cases where schools vetoed time off for training camps or competitions, it exclusively concerned courses and measures by the Turkish Football Federation. This could be explained by the fact that the Turkish Football Federation, in contrast to the DFB, does not offer school supervision for the players during the courses. 

However, some players reported that both the clubs and the YPC expressed a preference for the DFB—if the player had invitations from both associations. This is understandable to the extent that the club and the YPC are structurally anchored in the DFB. Furthermore, officials of the German club and the club’s YPC generally aim to recruit talented players independent of their parents’ nationality for the German national team.

“The [club and YPC] wanted me to play for Germany if I had both options.”

(Interviewee 1, Youth International DFB & TFF)

One player reported being invited to a meeting with club officials and representatives of the YPC in order to discuss the ‘national team question’.

“They called me into the office, talked to me about it and they had the opinion that I should play for the Germans. They said (-) at that moment they said we have two invitations, Germany, Turkey, you have to decide, but they told me: ‘Play for the Germans because with the Turks you’ll belong to the best’, he said. ‘And they’ll always invite you, even if you turn them down’, he said, ‘they’ll always invite you back because you’re one of the best or just the best with them.’ And they also wanted me to represent the [club name] with the DFB.”

(Interviewee 2, Youth International TFF)

The Turkish Football Federation is also a relevant corporate network actor for young athletes. The Turkish Federation is generally said to make great efforts to win over players with a Turkish background. The mother of one player, for example, reported the TFF had already made contact before the first invitation. In the course of this promotion, talks and meetings took place between the parents, the player and the European TFF scout at the time.

“Yes, there had already been contact before. Mr. Keser [then head of the European office of the Turkish Football Federation in Cologne] made a lot of effort to get in touch with him, he often called, we often met or just met and discussed the whole thing, how it is there and how it should work or what he is planning.”

(Interviewee 4, Youth International DFB & TFF)

Other players also reported that the federation regularly contacted the players personally and tried to convince them in personal conversations to pursue a national team career with the TFF.

“Constant calls from the [Turkish] federation and constant exchange with the coach. I had a lot of conversations there and that’s why I had a good feeling.”

(Interviewee 3, Youth International TFF)

The TFF’s strategy to influence the players by talking to their parents is appreciated by the players, not least because similar strategies of networking were not reported in connection with the DFB.

“At the federation’s office in Cologne there was a young woman who is also responsible for us, for the Turkish players, and she was always in touch. She called my parents. How I was doing. How they were doing. And I think that’s extremely strong, these actions. And they care. That’s the trust I mean. When I (-) I simply need this trust. When I know they’re behind me. They invite me. I’m in the national team, that makes me much stronger.”

(Interviewee 5, Youth International TFF)

In summary, it can be stated that the first invitation to a training course (sometimes only temporarily) changes a player’s network structures fundamentally. From the player’s point of view, the most favourable constellation is that both associations are interested in inviting him. In view of the fact that players at the youth level are not tied to a national association even if they play an international match for that association [39], the network structures are potentially volatile. However, dynamic changes of networks are not so much the result of the youth athletes’ personal decisions or preferences but are primarily driven by the structures of talent development. 

### 4.3. Players’ Agents

Players’ agents also play a relevant role in the decision-making process regarding the choice of a national team. The players unanimously reported that their agent was one of their first contacts regarding the national team issue.

“Well, I mostly talked to my agent about that kind of thing.”

(Interviewee 1, Youth International DFB & TFF)

Players’ agents play a special role in that they belong to the top sporting environment but are not formally integrated into the association’s structures of talent development. Given the fact that young athletes and advisors generally come into contact rather rarely and primarily in the context of career-relevant decisions, the connection has to be considered a weak tie, even though the players regard the relationship as important for their career development. Players’ agents serve the function of providing young athletes with knowledge and professional decision-making aids in career issues, as well as pointing out and opening up career options [44]. Based on the interviews, however, the players’ agents appear more as “supporters” in the players’ decision-making process. While they acknowledge the invitations from the top-level federations and appreciate them—similar to the club coaches—as a sports-related distinction, they try to remain neutral regarding the players’ decisions for a national team.

“I already had an agent back then. I just discussed it with him, what it looks like, what I should do. And he said: ‘Well, it’s a difficult decision. Do it the way you think is right for you, the way you feel comfortable.’”

(Interviewee 5, Youth International TFF)

This finding is particularly relevant as it refers to the conceptualisation of networks as “exchange systems” [26]. Surprisingly, the interviews do not provide any evidence that an exchange of relevant knowledge takes place between the players’ agents and the young athletes regarding specific decision-making aids that relate to their migrant backgrounds and chances to play for a national team. However, knowledge about the conditions of playing for a national team can be decisive for the careers of young athletes with a (Turkish) migrant background. While a migrant background can potentially open up multiple career options at the national team career level, the German Football Association, for example, makes German citizenship a condition, while citizenship is not a relevant selection criterion for the TFF. Not knowing about these connections is particularly consequential for those players who were born in Germany but had neither German nor dual citizenship. Players who only have Turkish citizenship are not eligible for the DFB national team. The interviews give reason to believe that this information was not available to the players concerned at the time of their first DFB invitation. From the player’s point of view, this proves precarious when players qualify for a DFB training course on sporting grounds, attend the course and are informed on site (regardless of their sporting performance) that they are not eligible to play for the DFB for reasons of citizenship and are consequently no longer considered for training courses.

### 4.4. Broker Positions and Structural Holes

Finally, we want to discuss the findings against the background of the theoretical concepts of “structural holes” and “brokers” [32]. Eliminating the lack of information on the part of the young athletes requires network experts who, on the one hand, have the corresponding expert knowledge and, on the other hand, make this knowledge available to the young athletes (in time). Such specific expertise cannot be expected from parents, family or friends. However, the interviews suggest that players neither receive information about citizenship-related recruitment conditions via strong network ties nor weak network ties. The fact that there is obviously a lack of actors who reliably ensure that the players have all the relevant information on the associations’ formal recruitment strategies is surprising, considering the high degree of professionalism in German top youth football. 

Particularly players’ agents should know about this problem as the lack of German citizenship eliminates the option of being considered in the German talent development system on a national team level. Against this background, the interviews draw attention to structural holes that prevent the exchange of relevant information, seeing that all the players we interviewed were born in Germany and would have had a good chance of acquiring German citizenship or dual citizenship under the current citizenship law. ‘Plugging’ these structural holes and setting up broker positions, therefore, seems appropriate not only to provide young athletes with a migrant background with a transparent basis for decision-making and to protect them from misunderstandings and disappointments, but also from a professional point of view, in order to avoid the DFB losing talents with a migrant background in the future.

## 5. Conclusions

Finally, it is important to discuss the limitations of our study. Firstly, we interviewed a comparatively small number of youth athletes only. There are several reasons for this: the nature of this study was mainly exploratory; and furthermore, recruiting youth international players means to address the absolute top performers in elite youth football, which represents a small minority of players only. The second limitation of our study is that we focused on youth players with a Turkish background only. However, since youth players with a Turkish background belong to the biggest ethnic minority group in Germany and are highly represented in German elite youth football, we decided to concentrate on this ethnic group in this explorative study. Concentrating on this ethnic group of elite youth football players significantly reduced the number of potential interviewees. In fact, the number of German-born international youth players with Turkish background training on YPC is generally very limited. Against the background that we could identify only 31 players overall who in principle matched the selection criteria, ten interviews with top young players appear to be a solid basis for a first exploratory analysis of this topic. Future studies should aim to include athletes with different migrant backgrounds.

A strength of our research design was that it allowed players to describe the decision-making process and the impact of network actors during this process retrospectively. This gave them the opportunity to reconstruct this process with a temporal distance. However, future studies that aim to document and analyse changes in athletes’ networks in more detail and at various stages of their careers should also consider using longitudinal designs.

Based on our findings, we can conclude that a successful career in elite sports requires athletes to make relevant decisions in the course of their careers, for which they depend on the support and advice of network actors in their surroundings. The results of the study confirm that the athletes do not act on their own but are surrounded by an environment of different actors who support them in the pursuit of sporting success [3]. The present analysis clearly shows that the most relevant networks for players with a migrant background regarding the decision to play for a national team are sport-related. Parents and family sometimes discuss the symbolic implications of the decision with regard to the parent’s country of birth, sometimes with regard to the players’ football career. Either way, families accepted the players as competent decision-makers in almost all cases and restricted themselves to the important role of emotional supporters in the athletes’ networks. However, it should be considered that the family environment can consist of different actors who sometimes give contradictory advice.

In the majority of cases, the young players’ networks are highly functionalised and provide the young athletes with information that leaves only limited room for manoeuvring. The association coaches have a particular influence on the players, which is probably due to the fact that they play a decisive role in the appointment of junior national players. Players’ agents occupy a structurally specific position in the networks. Even though players’ agents are marked as important supporters by the players, they do not play a particularly informative role with regard to the decision for a national team. Moreover, our interviews give reason to believe that sport-related networks of young elite football players have structural holes. For example, there is a lack of actors responsible for passing on information relevant to decision-making and ensuring that players are aware of all options and restrictions in the run-up to training courses organised by the top associations. With regard to the counselling of young talents with a migrant background, the interviews point to a considerable need for optimisation. In order to further professionalise talent promotion, but also to particularly support adolescent players with a migrant background, it is crucial to systematically install broker positions within the players’ networks. In this regard, it would be important to strategically decide where to install such broker positions within the system of talent promotion and how to qualify these brokers. The German YPCs have been constantly professionalised within the last years, and there is a lot of qualified staff available. These are good reasons to install such broker positions in this setting of talent promotion.

Taken together, our findings suggest that the decision-making process depends much less on the frequency of contact and the quality of relationships with other network actors than is assumed in network theories. Furthermore, the differentiation between weak and strong ties has little explanatory power with regard to sport-related decisions on the young elite level in football. On the contrary, weak ties can be even more influential on career-related decision-making processes than strong ties, not in the least because the biographically most relevant decisions by young elite players—from the players’ subjective points of view—are usually sport-related decisions. 

In the end, however, junior athletes filter, sort, prioritise and make sense of ‘irritations’ by their environment. In this sense, they do not regard themselves as passive transformers of network influences but autonomous decision-makers.

## Data Availability

No appliable.
